# Eosinophilic cystitis complicated with cystitis glandularis: a case report

**DOI:** 10.1186/s12894-022-01007-6

**Published:** 2022-04-09

**Authors:** Jun Bing Ye, Qian Chen, Ke Zeng, Xiao Bin Li, Ke Fei Deng, Jun Huang, Rong Chen

**Affiliations:** 1 Department of Urology, First People’s Hospital of Zigong City, Zigong, China; 2 Department of Nephrology, First People’s Hospital of Zigong City, Zigong, China

**Keywords:** Eosinophilic cystitis, Cystitis glandularis, Rare diseases, Case report

## Abstract

**Background:**

Eosinophilic cystitis (EC) is a rare inflammatory disease characterized by the gathering and infiltration of numerous eosinophilia (EOS) in the bladder wall. Because of Few cases of EC have been reported globally, the epidemiology of EC is not well known. We report herein the details of one very scarce case of large tumor-like eosinophilic cystitis complicated with cystitis glandularis (CG) diagnosed by biopsy.

**Case presentation:**

A 45-year-old Chinese man was referred to our hospital for the treatment of right lumbago and odynuria. Ultrasound examination indicated the low echo on the right portion wall and the neck of the bladder. Computed tomography showed a remarkable enhancing large mass that measured 5.0 cm × 2.3 cm located on the right portion of the bladder with undefined margin. For further treatment, diagnostic transurethral resection of the bladder was performed, the postoperative histopathological diagnosis was EC complicated with CG. After transurethral resection, antibiotics, glucocorticoids, and antihistamines were treated. The patient recovered uneventfully and was discharged on postoperative day 8 without evidence of recurrence followed-up for 6 months.

**Conclusion:**

Large tumor-like eosinophilic cystitis complicated with cystitis glandularis is rare, malignant tumors need to be ruled out. We deem that prompt biopsy led to the exact diagnosis, appropriate treatment led to better prognosis.

## Background

EC is a scarce inflammatory disease of the bladder, first presented in 1960 by Brown [1]. Because of the small number of patients suffer from EC, only about Two hundred cases of EC have been described, the etiology and pathogenesis of EC is not well understood, treatment modalities are not agreed upon. Most EC patients present with mucosal lesions of the bladder, only few studies have described mass-forming or malignancy-mimicking EC. As far as we know, there is no literature published about EC complicated with cystitis glandularis. The report of this case brings a guide for doctors to comprehend EC complicated with CG.

## Case presentation

**Fig. 1:**
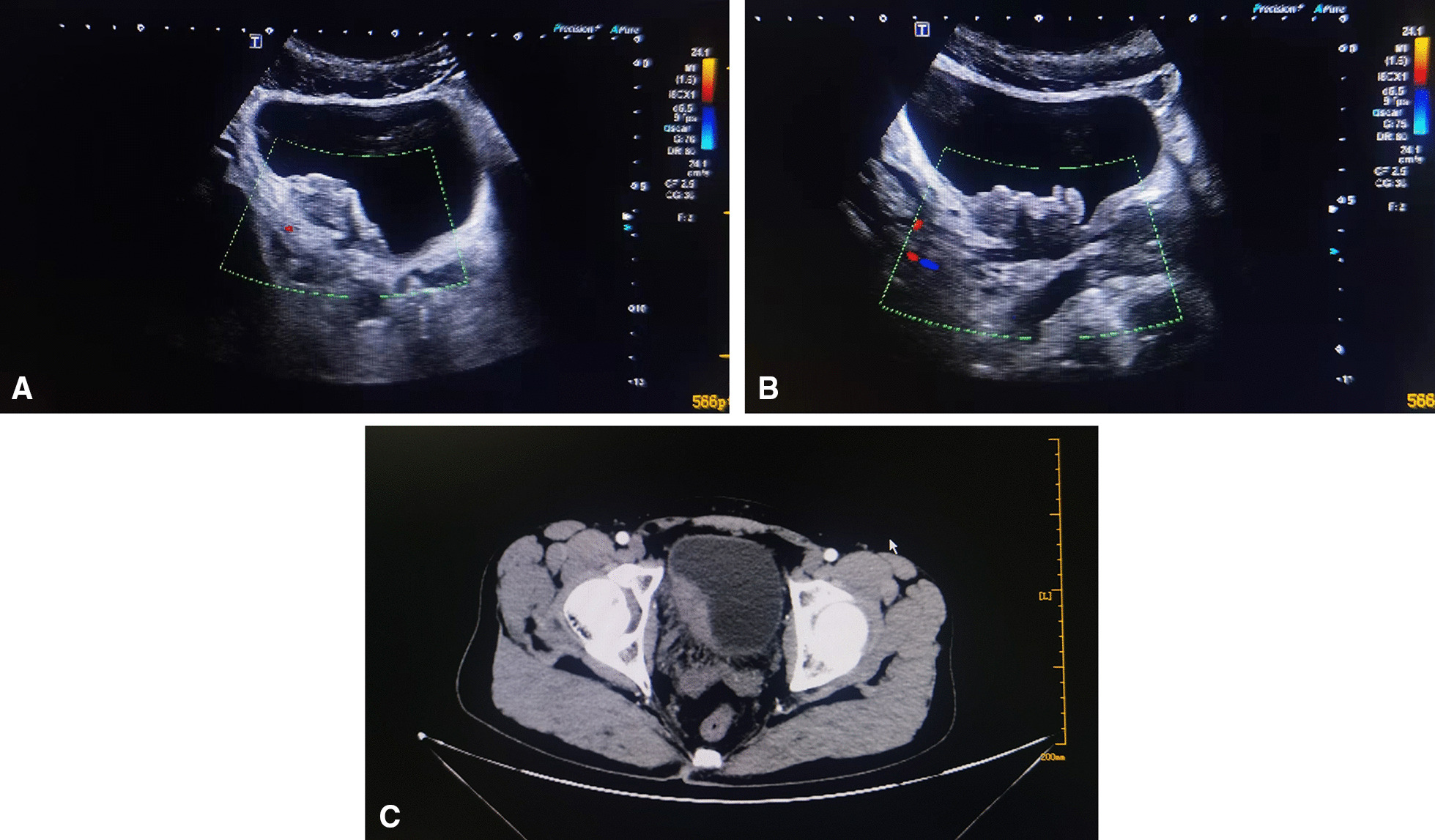
Ultrasound examination. **A** Low echo on the right portion wall of the bladder. **B** Low echo on the neck of the bladder; **C** a remarkable enhancing large mass on the right portion of the bladder with undefined margin

**Fig. 2:**
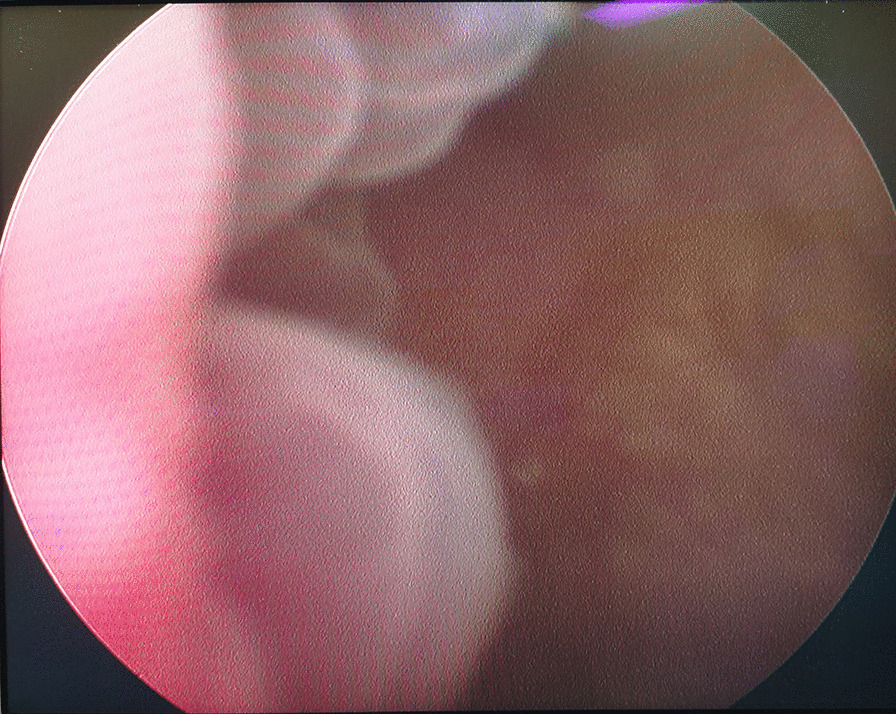
Cystoscopy showed a huge follicle-like mass lesion on the right portion wall and the neck of the bladder with a broad base

**Fig. 3:**
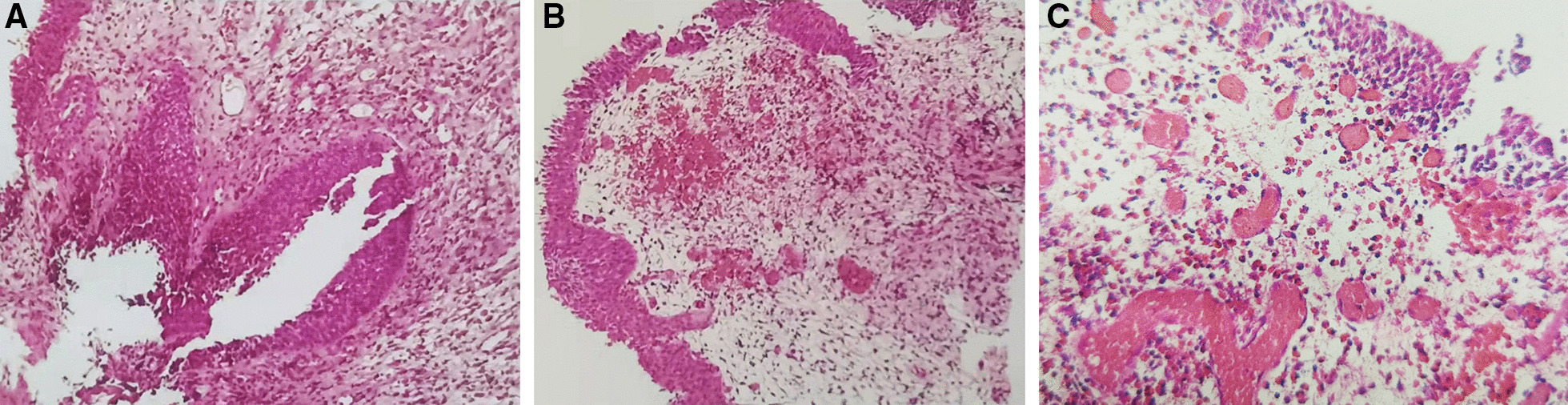
The histopathological diagnosis. **A**, **B** Cystoscopic biopsy affirmed the chronic mucosal inflammation of bladder; **C** numerous eosinophils per high power field of view and Brunn nests existed

A 45-year-old Chinese man was referred to our hospital for the treatment of right lumbago and odynuria, with no fever or gross hematuria. His past medical history was unremarkable and there was no history of any allergy or external trauma except a long working history in construction site. On admission, no abnormality was found in physical examination, Blood analyses revealed no leukocytosis (white blood cell count of 6.52 × 10^9^/L), no elevation of eosinophils (eosinophils count of 0.44 × 10^9^/L, eosinophil ratio Accounted for 6.7%). Renal function was normal (Serum creatinine level of 77.4 umol/L). Urinalysis confirmed no hematuria, leucocytes or protein. Ultrasound examination indicated the low echo on the right portion wall and the neck of the bladder, with dilation of the right lower ureter (Fig. 1A, B). Computed tomography showed no hydronephrsisa but a remarkable enhancing large mass that measured 5.0 cm × 2.3 cm located on the right portion of the bladder with undefined margin (Fig. 1C). Cystoscopy confirmed a huge follicle-like mass lesion on the right portion wall and the neck of the bladder with a broad base, in which blood vessels growing (Fig. 2). Cystoscopic biopsy affirmed the chronic mucosal inflammation of bladder (Fig. 3A, B). For further treatment, diagnostic transurethral resection of the bladder was performed, the postoperative histopathological diagnosis was EC complicated with CG (Fig. 3C). After transurethral resection, antibiotics, glucocorticoids, and antihistamines were treated, the catheter was indwelled continuously until postoperative day 6. The patient recovered uneventfully and was discharged on postoperative day 8. Cystoscopy and eosinophils levels of blood and urine were required to be examined every 3 months during the first year. There was no evidence of recurrence followed up for 6 months.

## Discussion and conclusion

In contrast with other cystitis, EC is a scarce inflammatory disease with low incidence, only about Two hundred cases of EC have been described globally. Among the 135 cases, the age of EC patients ranged from 5 to 87 years old, with an average age of 41.6 years old and no gender difference [2]. EC is thought to be associated with BRAFI463T gene mutation [3], which may lead to molecular defects of haematopoietic stem cells or myeloid cell. Sanaphylaxis caused by a variety of physical, chemical, biological and pathological factors is involved in the pathogenesis of EC, one-third of case’s cause still not found [4]. Such as parasites, dust, bladder injury, food and drug allergy, these antigens can cause an immunoglobulin E-mediated release of eosinophilic chemotactic factor which would ultimately lead to tissue damaged by releasing lysosomal enzymes and cytokines [5]. For the above reasons, we speculated that this patient was related to his long history of working in construction site. In addition, peripheral eosinophilia was not observed in this case, hypereosinophilia was not considered.

Peripheral eosinophilia is detected in about 43% of cases [2], more than half of cases without significantly elevation of peripheral eosinophilia. Eosinophils are rarely shed and degrade rapidly in urine, therefore urinalysis usually reveals proteinuria, microscopic haematuria, pyuria [6], only rare cases with eosinophiluria. Bladder irritation was the main manifestation of most EC patients, with frequency (67%) and dysuresia (62%) [2]. The main manifestation of our patient was right lumbago and odynuria, which may owning to the huge follicle-like mass lesion on the right portion wall and the neck of the bladder. It is suggested that not all bladder irritation or abnormal urinalysis could be found in EC cases. Imaging examinations are not helpful in determining those present as diffuse or irregular thickening of the bladder wall, or as nodular or huge masses. There was no characteristic manifestations with EC. Tumor-mimicking masses, polyps, follicles, ulcers, mucosal edema, hyperemia and erythema have all been reported. The large tumor-mimicking masses like our case was relatively rare and easily misdiagnosed. Eventually, pathological biopsy is needed, more than 25 eosinophils per high power field of view can be diagnosed with EC [7]. Significantly elevation of peripheral eosinophilia with the mucosa and submucosa was seriously involved in acute stage of EC, chronic mucosal inflammation was usually observed in chronic stage [8], which may the reason why cystoscopic biopsy affirmed the chronic mucosal inflammation of bladder with no eosinophilia founded in this patient.

Few literatures was reported on EC complicated with other disease. We report herein the case of large mass-forming EC complicated with CG diagnosed by biopsy. CG is a inflammatory disease with a low incidence, which characterized by the Brunn nests existed in the lamina propria of bladder stimulated by inflammation, obstruction and stone [9]. CG with large-area follicle-like and papillomatoid changes was classified as high-risk type, which with the risk of malignant transformation [10]. For patients with tumour-like masses, we speculate that EC and CG may mutually aggravate the progression of the disease, but whether EC would complicated with CG or malignancy tumor is unknown.

There is no consensus on the treatment of EC, medical treatments such as glucocorticoids, antihistamines, and antibiotics can be regarded as the initial treatment [11]. The recurrence of those patient who treated with medicine was 17%, whereas that was only 2.6% who treated with surgery [2]. For our patient, diagnostic transurethral resection of the bladder was performed, after that, antibiotics, glucocorticoids, and antihistamines were treated. Surgery is recommended for the treatment of those with large tumor-like or follicle-like lesions [10]. The patient recovered uneventfully without evidence of recurrence, which revealed that surgical treatment was preferred for large tumor-like EC complicated with CG.

Taken together, large tumor-like eosinophilic cystitis complicated with cystitis glandularis is rare, malignant tumors need to be ruled out. We deem that prompt biopsy led to the exact diagnosis, appropriate treatment led to better prognosis.

## Data Availability

All relevant data and materials are included in this article.

## Abbreviations

EC: Eosinophilic cystitis
EOS: Eosinophilia
CG: Cystitis glandularis
